# Morphological characterization of intraspecific variation for trichome traits in tomato (*Solanum lycopersicum*)

**DOI:** 10.1186/s40529-023-00370-3

**Published:** 2023-03-29

**Authors:** Satinderpal Kaur, Neetu Khanal, Robert Dearth, Rupesh Kariyat

**Affiliations:** 1 Department of Biology, University of Texas Rio Grande Valley, Edinburg, TX 78539 USA; 2 Department of Entomology and Plant Pathology, University of Arkansas, Fayetteville, AR USA

**Keywords:** Intraspecific variation, Trichome density, Solanum, Trichome sub-types, Dimensions

## Abstract

Trichomes, the hairlike protuberances in plants, have been well known to act as the first line of defense against herbivores, and abiotic stresses, along with other structural defenses such as spines, thorns, and waxes. We previously reported the tremendous variation in trichome traits among different wild and cultivated Solanum species and demonstrated that trichomes types and density are traditionally miscalculated and often misnamed. However, intraspecific variation in trichome traits is poorly understood, although this has implications for stress tolerance and resistance breeding programs in economically important crop species and can also mediate ecological interactions at multiple trophic levels in their wild congeners. In this study, using tomato as a model, we characterized the trichomes from 10 commonly grown varieties using a minimal sample prep desktop scanning electron microscopy, and followed up with estimating their dimensions across the varieties and trichome types. We hypothesized that although trichome number may vary, the varieties will have similar trichome types, based on current literature. Our results show that there is significant variation for trichome number as well as dimensions of trichome types among these varieties. Furthermore, when we separately analyzed the number and dimensions of commonly found glandular and non-glandular trichomes, the results were consistent with broad assessment of trichomes, showing consistent variation among varieties, suggesting that trichome studies should not be limited to basic classification into glandular and non-glandular, and should accommodate the sub-types and their dimensions.

**Supplementary Information:**

The online version contains supplementary material available at 10.1186/s40529-023-00370-3.

## Background

Plant structural defenses are morphological traits that deter herbivores from feeding on them (Boege and Marquis 2005). These includes spines, thorns, wax, trichomes and toughened leaves to name a few (Hanley et al. 2007). Among these, trichomes—the hairlike unicellular or multicellular epidermal appendages have been well studied for their roles in defense against biotic and abiotic stressors (Werker 2000). The development of trichomes on plant leaves start at a very early stage, even before the development of stomata (Werker 2000) and many genes have been identified, encoding for transcription factors which leads to expression of genes which consequently leads to the differentiation of trichomes from epidermis (Ishida et al. 2008; Tominaga-Wada et al. 2011). Trichomes have been primarily thought to be evolved against abiotic stress for temperature control by keeping the leaves cooler by decreasing the absorption of short-wave radiations (Gutschick 1999), water loss (Benz and Martin 2006) and photosynthetic conductance (Galmés et al. 2007; Morales et al. 2002). For example., *Arabidopsis* lines with higher trichome density were found to be resistant to UV-B radiations as compared to the mutants having lower trichome density showing their active role as UV protectant (Yan et al. 2012). More recently, their role as an anti-herbivore defense has been well documented in different study systems. These studies collectively show that they can have both pre and post ingestive effects and their roles can be affected by intrageneric variation, herbivore growth stages and feeding habits (Hanley et al. 2007; Løe et al. 2007; Kariyat et al. 2013, 2017; Watts and Kariyat 2021a, b).

Trichomes are found in a variety of shape, sizes and structures and are broadly classified into glandular and non-glandular based on the presence of a globular head (Werker 2000; Wagner et al. 2004). The non-glandular are usually unicellular and can be either unbranched or branched (Glas et al. 2012). On the other hand, glandular trichomes are multicellular and are reported to secrete a wide array of compounds ranging from attractants to toxic secondary metabolites (Schilmiller et al. 2008). Glandular trichomes have always been a topic of interest for plant biochemists owing to their ability to secrete compounds such as terpenes (Hallahan 2000), flavonoids (Kim et al. 2014), acyl sugars (Kroumova and Wagner 2003) and methylketones (Fridman et al. 2005) to name a few. Interestingly, most of these secondary metabolites are reported to act as anti-herbivore compounds. For example, in *Nicotiana attenuata,* the O-acyl sugars secreted by glandular trichomes impart a distinct odor to caterpillars which makes them more detectable by the predators (Weinhold and Baldwin 2011). Moreover, some of the metabolites from glandular trichomes are found to be of pharmaceutical importance, such as Artemisinin from *Artemisia annua* (Liu et al. 2011). Contrary to the vast roles of secretions of glandular trichomes, the non-glandular trichomes deter herbivory by restricting their feeding, movement, and oviposition (Tian et al. 2012; Dalin et al. 2008; Kariyat et al. 2019; Kaur and Kariyat 2020). Interestingly, in some cases, like in the genera Onopordum, Carthamus, and Gundelia that possess white and dense trichomes-they have been reported to mimic spider webs and mite nests which helps in deterring herbivory as herbivores tend to avoid spider webs (Yamazaki and Lev-Yadun 2015). However, for most trichome rich species, their morphological traits and potential pre and post ingestive roles have been less understood (but see Andama et al. 2020; Kaur and Kariyat 2020; Watts and Kariyat 2021a,b).

Trichomes are found to display tremendous variation in their density, types, shape, and sizes both interspecifically as well intraspecifically. Some examples of extensive trichome characterization done interspecifically are in potato (cultivated and wild species; Cho et al. 2017), water fern *Salvinia* (Barthlott et al. 2009), genus Capsicum (Kim et al. 2012), subfamily Faboideae (Leelavathi and Ramayya 1983), order polemoniales (Inamdar and Patel 1973), tribe Lactuceae (Krak and Mráz 2008), family compositae (Ramayya 1962), genus Convolvulus (Khokhar et al. 2012), order Malvales (Inamdar and Bhat 1983), genus Artemisia (Hayat et al. 2009), *Arabidopsis thaliana* (Bensch et al. 2009) and genus Solanum (Watts and Kariyat 2021a). On the other hand, by comparison, only a few studies have characterized the intraspecific morphological traits of trichomes, except for *Cucumis sativus* L., (Xue et al. 2019), *Datura wrightii* (Van Dam et al. 1999) and *Withania somnifera* (Munien et al. 2015). While intraspecific variation is less studied, we recently documented that within a leaf, abaxial and adaxial surface have consistent variation in trichome density in Solanum genus, with consequences for defense against a specialist herbivore (Watts and Kariyat 2021b). Collectively, these studies show the importance of understanding the morphological and functional diversity of trichomes at genus, species, and even between abaxial and adaxial leaf surfaces (Watts and Kariyat 2021b).

Solanaceae is one of the largest families in Angiosperms, with a diverse membership based on their habitat, economic and ecological importance, and human use as food, medicine, as well as weeds, and ornamentals (Knapp et al. 2004; Gebhardt 2016). Because of their diversity within the family, Solanaceae, and Solanum genus is commonly used as a model for study of trichomes: morphology and function (Bar and Shtein 2019; Watts and Kariyat 2021a, b; Watts and Kariyat 2022). Solanaceae family members have also been used as a model to understand trichome X herbivory interactions. (Hill et al. 1997; Peiffer et al. 2009; Tian et al. 2012; Oney and Bingham 2014; Murungi et al. 2016; Kariyat et al. 2013; 2017, 2018; Watts and Kariyat 2022). Tomato (*Solanum lycopersicum)* possibly the most economically important species in the family has been documented to possess diverse subtypes of glandular trichomes (Kang et al. 2010a), and had been used as a model for deciphering the role of secretions from glandular trichomes in relation of herbivore defense (Li et al. 2004; Boughton et al. 2005; Van Schie et al. 2007; Kang et al. 2010b), and they have also been found to detoxify heavy metals such as cadmium, nickel, and zinc (Koul et al. 2021). The cultivated species of Solanaceae such as tomato and tobacco (*Nicotiana tabacum)* are found to have dense glandular trichomes. On the other hand, invasive weed species such as silverleaf nightshade (*Solanum eleaegnifolium;* Petanidou et al. 2018) and noxious weeds such as horsenettle (*Solanum carolinense*) tend to have denser non-glandular trichomes (Kariyat et al. 2018; Watts and Kariyat 2021a). However, most studies have either resorted to pairwise comparisons, or pausing at trichome morphology traits ignoring trichome subtypes or have failed to acknowledge their branching patterns (stellate) and dimensions.

Evaluating the intraspecific variations in trichome density and dimensions can prove to be a valuable tool to develop better defended plants to continuously evolving biotic stresses. Since trichomes have both pre and post ingestive impacts on herbivores, this can be a possible target for resistance breeding programs. Keeping this in mind, by using the trichome classification provided by (Roe 1971), glossary provided by (Payne 1978) and terminology, trichome characterization, and graphics by Watts and Kariyat 2021a), we did a detailed examination of the trichomes of ten important tomato varieties grown in United States. We then used Shannon Diversity index to estimate the trichome richness and abundance in each of the ten varieties. The following questions were asked:

1. Is there variation of trichome density and dimensions within the common varieties of tomato?
2. What are the most common glandular and non-glandular trichomes in tomato?
3. Is there variation in density and dimensions of the most common trichomes among these varieties. We hypothesized that although trichome density may vary, the varieties will have similar trichome types and dimensions, based on current literature on tomato trichomes.

## Materials and methods

### Plant materials

Ten commonly cultivated varieties of tomato, *Solanum lycopersicum*, namely, Big beef, Black prince, Supersweet, Tidy treats, Pink tiger, Sungold, Pink berkeley, Purple bumble bee, Indigo cherry drops and Black krim were used for this experiment. Due to the extensive use of scanning electron microscopy required, we had to limit the varieties to 10 from a long list of all commercial varieties. These seeds were bought from Johnny’s selected seeds (Johnnyseeds.com).

The seeds were sown in the trays (21.06″ × 10.81″ × 2.5″), filled with potting mixture (Berger BM 6HP, which is a peat and perlite mix having incubated pH of 5.4–6.2). The trays were kept in controlled conditions (26 °C temperature, 18: 6 light: dark cycle). Seedlings at 2 true-leaf stage were then transplanted in the same potting mixture, same environmental conditions but in separate pots (3″ × 3″ × 2.5″). The plants were applied with fish fertilizer (Alaska Fish Emulsion Plant Food, 5-1-1 Fertilizer, Kwik Retail LLC), 15 days after transplanting with irrigation water and were watered regularly.

### Electron microscopy

Electron microscopy was done on 9 fully developed leaves taken from 3 randomly selected plants from each variety. For this, a desktop scanning electron microscope (DSEM; SNE-4500 Plus Tabletop Nanoimages LL, Pleasanton, CA, USA) was used to capture images from adaxial surfaces of leaf samples. The leaves were used fresh without any sputter coating, or any chemical treatment and fresh leaves were cut into a rectangular portion from middle with the help of scissors and mounted on the aluminum stub by using double sided carbon tape (Watts et al. 2022). This methodology allowed us to do minimum sample preparation for imaging, but also potentially avoided any artifacts during sample prep that tends to take much longer with traditional SEM (Watts et al. 2022).

### Characterization of trichomes

To assess the morphology of trichomes, images were taken at varied magnifications, mostly from 100×–2000×, to get maximum resolution. Moreover, images of certain trichomes were captured at different angles to get more specific details. The trichomes were not only classified into glandular and non-glandular, but more finely into sub-types based on the classification given by Watts and Kariyat 2021a. The glandular trichomes were mainly classified according to the shape and structure of their glandular head.

**Fig. 1:**
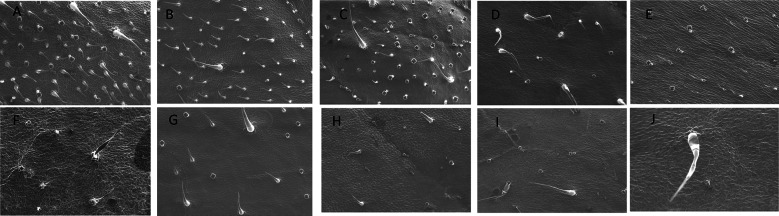
Scanning electron microscope (SEM) images captured at 100× of 10 varieties of tomato, *Solanum lycopersicum* (A, Sungold; B, Supersweet; C, Pink tiger; D, Black krim; E, Big beef; F, Pink berkeley; G, Tidy treats; H, Purple bumble bee; I, Indigo cherry drops; J, Black prince)

After imaging, the trichome density of these varieties was assessed, by capturing the images at 100× magnification (Fig. 1). The magnification was kept consistent among all the leaf samples. By using “Nanoeye” software in DSEM, we measured the surface area at 100× (translates to 1.89 mm^2^). The number of trichomes were counted separately according to their classification, and the trichome density per mm^2^ was calculated using the equation (Watts and Kariyat 2021a):

$$ {\text{Trichome}}\,{\text{density}}\,\left( {1\,{\text{mm}}^{2} } \right) = \frac{{{\text{number}}\,{\text{of }}\,{\text{trichomes }}\,{\text{in }}\,{\text{the }}\,{\text{image}}\,{\text{captured }}\,{\text{at }}\,100\, \times }}{1.89} $$

To quantify the variation in trichome dimensions, we also captured the images (9 leaves per variety) using a feature “straight line” in “Nanoeye” software. For non-glandular trichomes, length of trichomes was measured. For the glandular trichomes with large quadricellular head and globular head, attenuate glandular trichome with small glandular head, the diameter of head was measured. And for all other glandular trichomes, the length data was collected.

### Statistical analysis

Trichome density of all the different categories of trichomes were calculated for ten varieties along with their mean ± standard error. The number of trichomes in different varieties was analyzed first through the broad categories (number of total trichomes, number of glandular trichomes and number of non-glandular trichome). Moreover, we selected two most found glandular trichomes among these varieties and analyzed them separately. Since, the non-glandular trichomes were only three, we analyzed them all separately. Generalized regression with Poisson distribution was used to analyze the number of trichomes of all categories. Similarly, generalized regression with Poisson distribution was used to analyze the diameters of glandular head of two most common glandular trichomes found and length of non-glandular trichomes. Tukey’s post hoc tests were also done for doing pairwise comparisons for all the above-mentioned tests. All analyses were carried out using JMP Pro 15 (SAS Institute Inc, Cary, NC).

### Shannon–Weiner diversity index

The Shannon–Weiner diversity index was used to test the diversity of trichomes in the different varieties of tomato. The diversity index was computed using the formula below:

$$ H^{\prime} = - \sum Pi Ln Pi,\,\,{\text{in}}\,\,{\text{which}}\,\,Pi = \frac{ni}{N} $$

Here, $H^{\prime}$ = Shannon diversity index; Pi = Relative number of trichomes; $ni$ = trichome number *i*; N = total number of trichomes.

## Results

**Fig. 2:**
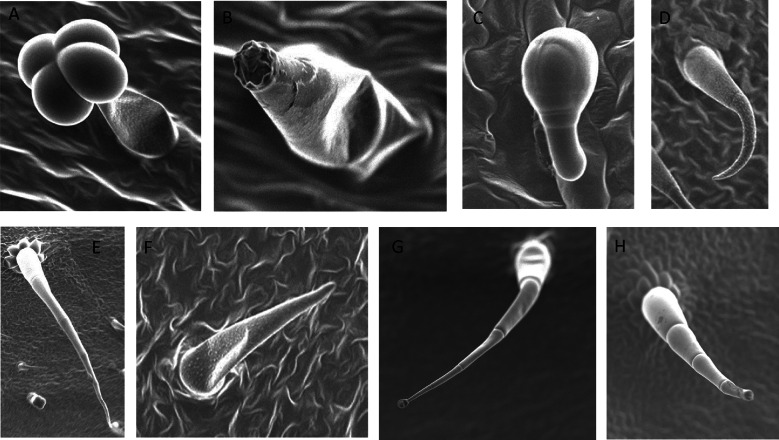
The major types of glandular trichomes found in tomato. **A** Glandular trichome with large quadricellular head; **B** Attenuate glandular hair with small glandular tip; **C** Glandular hair with large globular head; and non- glandular trichomes found; **D** Crescent non-glandular with multicellular jointed stalk; **E** Subulate non-glandular hair with multicellular jointed stalk, multicellular base; **F** Subulate non-glandular hair with pulvinate base and pedestal; Some other glandular trichomes; **G** Hooked subulate glandular hair with multicellular jointed stalk and small glandular tip; **H** Subulate glandular hair with multicellular jointed stalk, multicellular base, small glandular tip. Images captured in Scanning electron microscope at magnification 200–1000×

Broadly, the trichomes present on *Solanum lycopersicum* varieties were categorized as glandular and non-glandular trichomes. These were further divided into separate subtypes based on fine differences in their morphology. For glandular trichomes, the shape and structure of their glandular head was the major factor in characterization, while for the non-glandular trichomes, the number of joints of their stalks was considered as a factor for classification. The information on the types of trichomes present along with their pictorial representation is provided in Additional file 1: Table S1. The glandular trichomes were found more commonly than the non-glandular trichomes in all the varieties. A total of 9 trichome types were found in which 6 were glandular and 3 being non-glandular. Most of the glandular trichomes are single stalked having a glandular head of varied shapes and non-glandular trichomes found are long and attenuate with varying number of jointed stalks and base cellularity (for detailed nomenclature see Watts and Kariyat 2021a). More interestingly, out of these 9, 2 glandular trichomes (glandular hair with large quadricellular head and attenuate glandular hair with small glandular tip) and 1 non-glandular trichome (crescent non-glandular with multicellular jointed stalk) were found in all the 10 tested varieties. However, one trichome out of all 9, acuminate glandular hair with bicellular stalk and small glandular tip was rare and only present in two of the ten tested varieties. The mean trichome density ± standard error of the trichomes (Table 1) was calculated separately for all varieties except for the rare trichomes (those present only in 2 or less out of 9 tested leaf samples). Clearly, while trichome types shows variation among the varieties, 2 glandular and one non-glandular non stellate sub type was consistently present in all the varieties, suggesting conserved functional consequences (Fig. 2).

**Table 1:** The density and dimensions (in µm) of trichomes in ten varieties of tomato

Variety	Trichomes	Mean density	SE	Mean dimensions (in µm) average	SE
BLACK KRIM	Hooked subulate glandular hair with multicellular jointed stalk and small glandular tip	0.53	0	396.9	84.1
	Glandular hair with large globular head	2.37	1.31	Data not available	
	Glandular hair with large quadricellular head	4.018	1.23	54.2	2.14
	Attenuate glandular hair with small glandular tip	1.612	0.196	48.8	
	Subulate glandular hair with multicellular jointed stalk, multicellular base, small glandular tip	1.055	0.303	413.8	106.3
	Subulate non-glandular hair with pulvinate base and pedestal	1.67	0.28	139.9	15.76
	Crescent non-glandular with multicellular jointed stalk	3.48	1.29	321.27	42.87
	Subulate non-glandular hair with multicellular jointed stalk, multicellular base and distinct subsidiary cells	4.214	2.1	436.4	30.3
PINK BERKELEY	Hooked subulate glandular hair with multicellular jointed stalk and small glandular tip	1.58		491.8	53.03
	Glandular hair with large globular head	0.53		Data not available	
	Glandular hair with large quadricellular head	2.87	0.41	56.4	2.38
	Attenuate glandular hair with small glandular tip	1.06		60	
	Subulate glandular hair with multicellular jointed stalk, multicellular base, small glandular tip	1.14	0.16	553.33	
	Subulate non-glandular hair with pulvinate base and pedestal	1.85	0.34	348.9	68.5
	Crescent non-glandular with multicellular jointed stalk	1.84	0.26	339.2	49.28
BIG BEEF	Glandular hair with large globular head	2.21	0.63	33.83	2.7
	Glandular hair with large quadricellular head	2.03	0.72	60.29	4.36
	Attenuate glandular hair with small glandular tip	2.01	0.42	59.04	4.87
	Subulate glandular hair with multicellular jointed stalk, multicellular base, small glandular tip	1.06		410.5	
	Subulate non-glandular hair with pulvinate base and pedestal	1.85	0.97	204.4	39.14
	Crescent non-glandular with multicellular jointed stalk	3.27	0.35	164.875	14.31
	Subulate non-glandular hair with multicellular jointed stalk, multicellular base and distinct subsidiary cells	0.53		547.5	176.5
BLACK PRINCE	Hooked subulate glandular hair with multicellular jointed stalk and small glandular tip	0.53		Data not available	
	Glandular hair with large globular head	1.05	0.52	33.4	2.4
	Glandular hair with large quadricellular head	0.635	0.1	72.53	5.54
	Attenuate glandular hair with small glandular tip	1.497	0.46	85.2	4.88
	Subulate glandular hair with multicellular jointed stalk, multicellular base, small glandular tip	1.06		210	
	Subulate non-glandular hair with pulvinate base and pedestal	0.88	0.176	341.5	221.5
	Crescent non-glandular with multicellular jointed stalk	1.434	0.47	254.2	59.29
	Subulate non-glandular hair with multicellular jointed stalk, multicellular base and distinct subsidiary cells	1.06	0.303	550.5	96.31
PURPLE BUMBLE BEE	Hooked subulate glandular hair with multicellular jointed stalk and small glandular tip	0.53		697	
	Glandular hair with large globular head	2.24	0.75	30.9	5.39
	Glandular hair with large quadricellular head	1.41	0.32	72.93	3.87
	Attenuate glandular hair with small glandular tip	1.98	0.33	46.8	3.6
	Subulate glandular hair with multicellular jointed stalk, multicellular base, small glandular tip	0.53		697	
	Subulate non-glandular hair with pulvinate base and pedestal	0.88	0.35	193	
	Crescent non-glandular with multicellular jointed stalk	1.37	0.35	382.7	85.99
	Subulate non-glandular hair with multicellular jointed stalk, multicellular base and distinct subsidiary cells	1.58		265.21	49.85
TIDY TREATS	Glandular hair with large quadricellular head	1.94	0.44	57.55	2.61
	Attenuate glandular hair with small glandular tip	0.924	0.25	56.17	3.65
	Acuminate glandular hair with bicellular stalk and small glandular tip	3.7		84	2.64
	Subulate non-glandular hair with pulvinate base and pedestal	2.38		113.63	16.53
	Crescent non-glandular with multicellular jointed stalk	2.72	0.66	222.7	32.2
	Subulate non-glandular hair with multicellular jointed stalk, multicellular base and distinct subsidiary cells	0.53		258.76	36.27
INDIGO CHERRY DROPS	Glandular hair with large quadricellular head	1,322	0.37	42.9	2.11
	Attenuate glandular hair with small glandular tip	0.88	0.35	64.1	6.74
	Glandular hair with globular head	0.53		Data not available	
	Subulate glandular hair with multicellular jointed stalk, multicellular base, small glandular tip	0.53		625	
	Crescent non-glandular with multicellular jointed stalk	1.054		498.71	112.95
	Subulate non-glandular hair with multicellular jointed stalk, multicellular base and distinct subsidiary cells	1.408	0.4	534.47	34.96
SUNGOLD	Hooked subulate glandular hair with multicellular jointed stalk and small glandular tip	0.53		148	38
	Glandular hair with large quadricellular head	4.93	1.101	57.5	1.88
	Attenuate glandular hair with small glandular tip	2.43	1.39	61.87	4.14
	Acuminate glandular hair with bicellular stalk and small glandular tip	0.53		Data not available	
	Glandular hair with globular head	1.055	0.52	Data not available	
	Subulate glandular hair with multicellular jointed stalk, multicellular base, small glandular tip	1.056	0.303	555.25	68.5
	Subulate non-glandular hair with pulvinate base and pedestal	1.585	1.055	164.52	16.36
	Crescent non-glandular with multicellular jointed stalk	15.69	4.66	321.933	77.12
	Subulate non-glandular hair with multicellular jointed stalk, multicellular base and distinct subsidiary cells	1.58		370.2	60.21
SUPERSWEET	Glandular hair with large globular head	1.76	0.46	Data not available	
	Glandular hair with large quadricellular head	2.77	0.63	45.03	1.56
	Attenuate glandular hair with small glandular tip	1.232	0.17	56.95	3.05
	Subulate glandular hair with multicellular jointed stalk, multicellular base, small glandular tip	0.794	0.26	399.5	
	Crescent non-glandular with multicellular jointed stalk	13.9	2.36	265.93	26.9
	Subulate non-glandular hair with multicellular jointed stalk, multicellular base and distinct subsidiary cells	1.32	0.29	310.2	23.8
PINK TIGER	Glandular hair with large globular head	2.633	2.1	Data not available	
	Glandular hair with large quadricellular head	5.69	3.83	60.203	1.99
	Attenuate glandular hair with small glandular tip	2.2	0.66	42.91	4.01
	Subulate non-glandular hair with pulvinate base and pedestal	1.055	0.52	187.52	27.56
	Crescent non-glandular with multicellular jointed stalk	2.64	1.27	181.01	24.4
	Subulate non-glandular hair with multicellular jointed stalk, multicellular base and distinct subsidiary cells	0.53	0	226.2	29.64

The dimensions of glandular trichome with globular head, large quadricellular head and attenuate hair with small glandular tip was represented by width of glandular head, while for all other types, length of trichome was measured. The trichomes whose standard error is not mentioned, were rarely found

### Number of trichomes

**Fig. 3:**
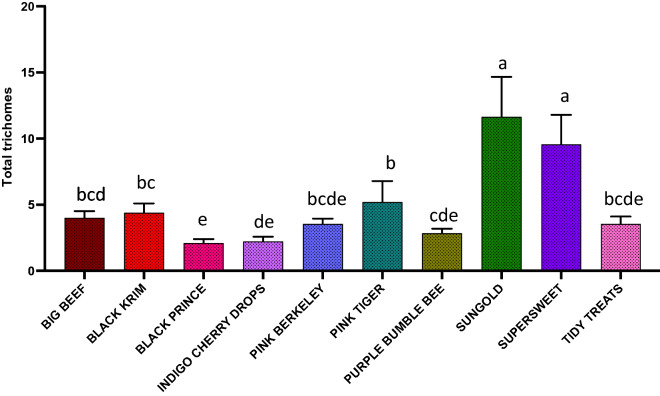
The comparison of total number of trichomes present in ten varieties of tomato. Detailed statistics are presented in Table 2

**Fig. 4:**
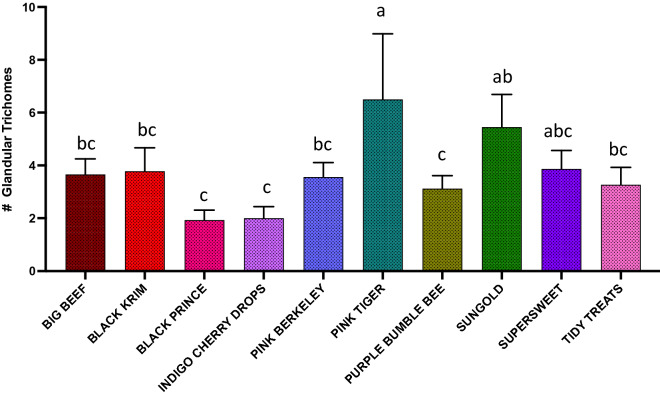
The comparison of total number of glandular trichomes present in ten varieties of tomato

**Fig. 5:**
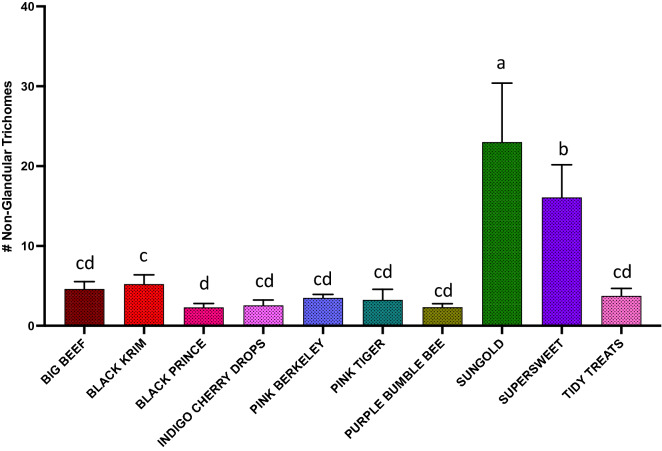
The comparison of total number of non-glandular trichomes present in ten varieties of tomato

**Fig. 6:**
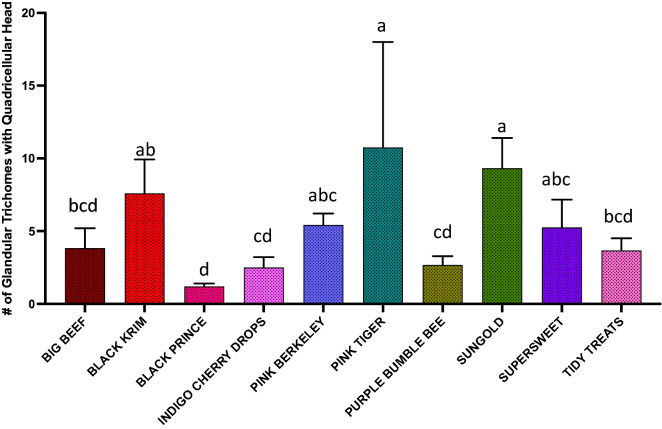
The comparison of total number of glandular trichome with large quadricellular head (which is most common among varieties) present in ten varieties of tomato

**Fig. 7:**
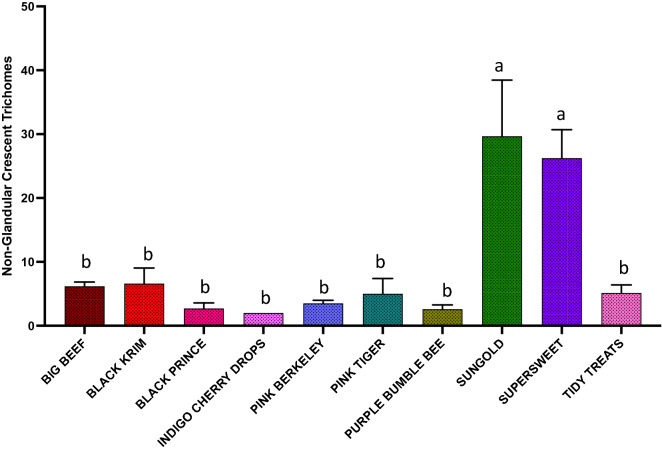
The comparison of total number of the most common non-glandular trichome (crescent non-glandular with multi-cellular jointed stalk) trichomes present in ten varieties of tomato

**Fig. 8:**
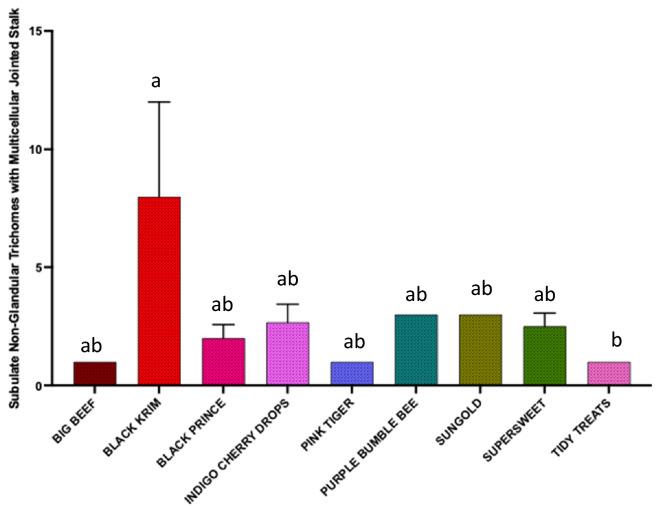
The comparison of total number of subulate non-glandular hair with multicellular stalk, multicellular base, and distinct subsidiary cells trichome present in ten varieties of tomato

The results of generalized regression showed that the number of total trichomes varied significantly among the varieties (Fig. 3; Wald Chi-Square = 460.07, *p* < 0.0001), as expected. moreover, the number of glandular (Fig. 4; Wald Chi-square = 62.83, *p* < 0.0001) trichomes also varied significantly among the varieties, along with number of non-glandular trichomes (Fig. 5; Wald Chi-square = 538.8, *p* < 0.0001). Interestingly, when we ran separate analysis for the most commonly found glandular trichome (glandular hair with large quadricellular head), the number was significantly different (Fig. 6; Wald Chi-square = 81.71, *p* < 0.0001). The number of second common glandular trichome also varied significantly among varieties (Wald Chi-square = 13.21, *p* = 0.1531). Similarly, we separately analyzed all the three non-glandular trichomes, crescent non-glandular with multicellular jointed stalk (Fig. 7; Wald Chi-square = 399.39, *p* < 0.0001), subulate non-glandular hair with multicellular jointed stalk, multicellular base, and distinct subsidiary cells (Fig. 8; Wald Chi-square = 25.53, *p* = 0.013), but the number of third non-glandular trichome, was found to be non-significant having (Wald Chi-square = 6.283, *p* = 0.5071). Taken together, our results show that the non-glandular trichome density varied more among the tested varieties (for more details of trichome number analysis and statistics, see Table 2).

**Table 2:** Results of Generalized linear regression with Poisson distribution of the number and dimensions of trichomes

Source	DF	Wald Chi-square	*p*-value
Total number of trichomes	9	460.07	**<0.0001**
Number of glandular trichomes	9	62.83	**<0.0001**
Number of non-glandular trichomes	9	538.8	**<0.0001**
Number of glandular trichomes with quadricellular head	9	81.71	**<0.0001**
Number of attenuate glandular hairs with small head	9	13.21	0.1531
Number of crescent non-glandular trichomes	9	399.39	**<0.0001**
Number of subulate non-glandular trichomes with multicellular stalk	8	25.53	**0.0013**
Number of subulate non-glandular trichomes with pulvinate base	7	6.283	0.5071
Width of glandular trichomes with quadricellular head	9	188.53	**<0.0001**
Diameter of attenuate glandular hairs with small head	9	97.15	**<0.0001**
Length of crescent non-glandular trichomes	9	2271.69	**<0.0001**
Length of subulate non-glandular trichomes with multicellular stalk	8	6264.64	**<0.0001**
Length of subulate non-glandular trichomes with pulvinate base	7	1409.16	**<0.0001**

Bold values indicate statistically significant *p* values

### Trichome dimensions

**Fig. 9:**
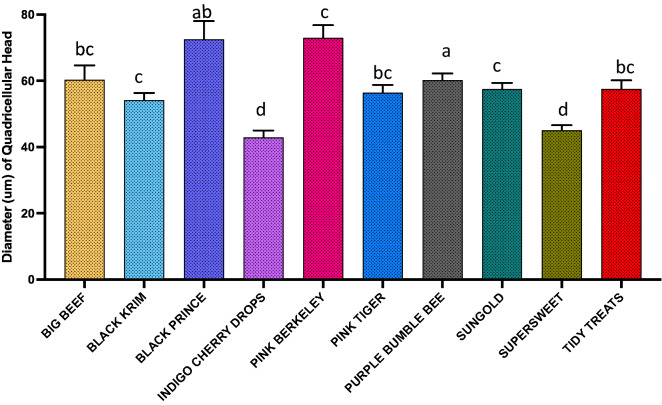
Comparison of diameter (µm) of quadricellular head of glandular trichome with large quadricellular head in ten varieties of tomato

**Fig. 10:**
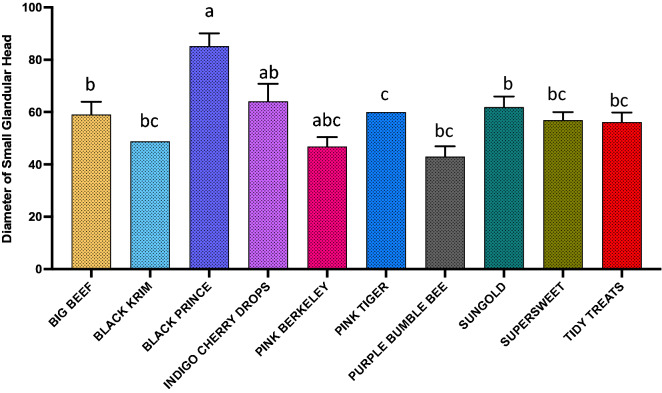
Comparison of diameter (µm) of small glandular head of attenuate glandular hair with small glandular tip trichome among the ten varieties of tomato

**Fig. 11:**
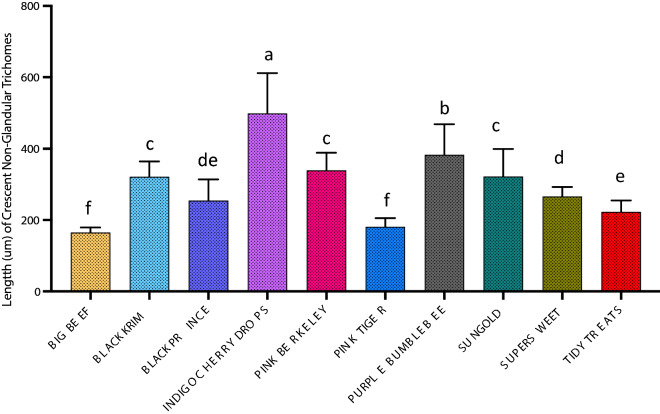
Comparison of length (µm) of most common non-glandular trichome (crescent non-glandular with multicellular jointed stalk) in ten varieties of tomato

**Fig. 12:**
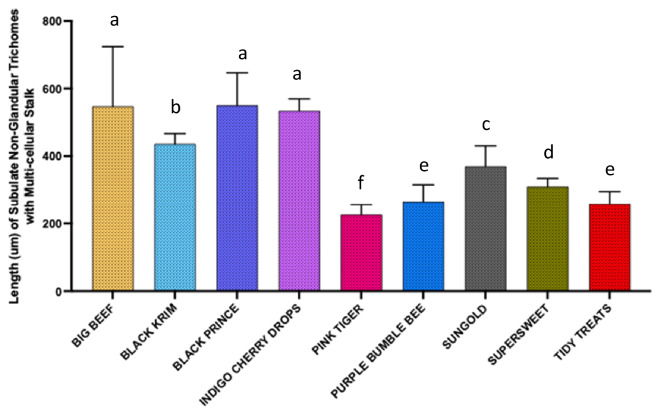
Comparison of length (µm) of second common non-glandular trichome (subulate non-glandular hair with multicellular jointed stalk, multicellular base, and distinct subsidiary cells) in ten varieties of tomato

**Fig. 13:**
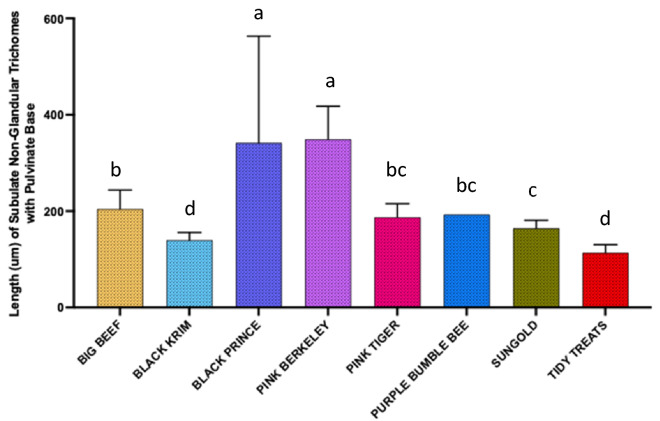
The comparison of length (µm) of third non-glandular trichome (subulate non-glandular hair with pulvinate base and pedestal) for ten varieties of tomato

For dimension analyses of glandular trichomes, we measured the width of the quadricellular head of most common glandular trichome and width of the small glandular head of second common trichome, and for non-glandular trichomes, the length of all three non-glandular trichomes was measured. For the glandular trichome with large quadricellular head, the width of the head was measured, and was found to be significantly different among varieties (Fig. 9; Wald Chi-square = 188.53, *p* < 0.0001), a similar trend was also found with the second most common trichome, attenuate glandular hair with small glandular tip, in which the diameter of small glandular tip varied significantly (Fig. 10; Wald Chi-square = 97.15, *p* < 0.0001). For non-glandular trichomes, the length of the trichomes was analyzed for the most common trichome, the crescent non-glandular with multicellular jointed stalk, and it varied significantly (Fig. 11; Wald Chi-square = 2271.69, *p* < 0.0001). And for the second trichome, the subulate non-glandular hair with multicellular jointed stalk, multicellular base and distinct subsidiary cells, the difference in length was significant (Fig. 12; Wald Chi-square = 6264.64, *p* < 0.0001). A similar trend was also observed for the third non-glandular trichome, subulate non-glandular hair with pulvinate base and pedestal, (Fig. 13; Wald Chi-square = 1409.16, *p* < 0.0001). Our results show that like trichome density, trichome dimensions also show more variation for non-glandular trichomes among the 10 tested varieties (see Table 3 for more detailed statistics and analysis of trichome dimensions).

**Table 3:** The results of Shannon Weiner diversity index and equitability for trichomes in tomato varieties

Varieties	Shannon diversity index	Equatibility
Black krim	1.834	0.880
Pink berkeley	1.450	0.744
Big beef	1.712	0.860
Black prince	1.806	0.847
Purple bumble bee	1.758	0.846
Tidy treats	1.507	0.841
Indigo cherry drops	1.328	0.741
Sungold	1.012	0.461
Supersweet	0.919	0.513
Pink tiger	1.451	0.810

### Shannon–Weiner diversity index

We used Shannon–Weiner diversity index as an indicator for measuring trichome diversity and found that the maximum diversity index equitability in variety Black krim (0.88). Other varieties having equitability more than 0.8 are Purple bumble bee, Black prince, Big beef, Pink tiger, and Tidy treats, whereas the minimum equitability was found in varieties Sungold and Supersweet, which was 0.46 and 0.51 respectively. This shows that in spite of having first and second most maximum trichome density, the diversity of trichomes is low in these varieties as compared to other varieties. The equitability of all other varieties was in the range of 0.7–0.88, which indicates that these varieties are in general, diverse in their trichomes (Table 3).

## Discussion

To examine whether intraspecific variation through domestication affects defense traits, we examined in detail, the trichomes of 10 commonly cultivated tomato varieties. We found that there is a tremendous variation in trichome morphology, type, density as well as dimensions among these varieties, possibly for the first time. We also found that the density of glandular trichomes is higher than non-glandular trichomes. Also, among glandular trichomes, subtypes were found based on the shape and structure of their glandular head. However, the small glandular hair with large quadricellular head is most common which was found in all the varieties. Our detailed examination adds another line of evidence for designing trichome studies to consider this variation in trichome traits (Watts and Kariyat 2021a).

Our results are also consistent with the results of Van Dam et al. (1999), who found significant variation in the phenotype of trichomes among the populations of *Datura wrightii*, another species in Solanaceae, commonly used in ecological studies (Nunez-Farfan and Dirzo 1994; Valverde et al. 2001). And we recently (Watts and Kariyat 2021a) documented and described a well-defined classification of trichome types from 14 wild and cultivated species of Solanum genus. However, since, of all the cultivated Solanum species, tomato is arguably, the most important species, and this study revealed additional results including the presence of rare glandular types trichome type (Acuminate glandular hair with bicellular stalk and small glandular tip) in a few varieties.

Out of all the 9 types of trichomes found in tomato, 6 were glandular trichomes. The glandular trichomes are reported to secrete many volatile compounds which are known to trigger insecticidal effects in the plants. For example, the tomato mutant having very less glandular trichomes with quadricellular head was found to accumulate lower amount of monoterpenes, sesquiterpenes, and flavonoids. Consequently, this mutant was highly susceptible to the herbivores in natural conditions, suggesting the anti-herbivore nature of these trichome secreted compounds (Kang Liu et al. 2010). Many scientists have done the gene expression profiling of trichomes (Cui et al. 2011) and transcriptomics (Balcke et al. 2017). Our study showed the enormous variation in the density of this trichome along with diameter of glandular quadricellular head and can act as a foundation to decipher the function of its size and density on the plant defense as this trichome secretes many important compounds, possibly involved in plant defense against herbivores.

Contrary to the glandular trichomes, the non-glandular trichomes are documented to affect feeding and oviposition of herbivores because of their structure. For example, Tian et al. 2012 found that in tomato, the mutants with high density of non-glandular trichomes negatively affect the feeding and growth of Colorado potato beetle, *Leptinotarsa decemlineata.* Apart from this, the non-glandular trichomes are found to cause post-ingestive damage by rupturing the peritrophic gut membrane of caterpillars (Kariyat et al. 2017). Similarly, in rice the silicified non-glandular trichomes were found to damage the gut membranes of rice chewing herbivores and thus acting as an important defense component (Andama et al. 2020). In this study, we examined thoroughly the length variations of important non-glandular trichomes of tomato and found that they significantly differ from each other-suggesting potential functional consequences for both settling and feeding disruptions (Kariyat et al. 2018), and post ingestive effects on the gut lining (Kariyat et al. 2017). Clearly, a detailed examination of diets supplemented with trichomes from these varieties, and their effects on herbivore fitness is warranted.

Trichomes, in general, have been very well studied for their role in plant defense against herbivores (Kariyat et al. 2017, 2019; Watts and Kariyat 2021b). They are also highly inducible through herbivory (Kariyat et al. 2013; Traw and Dawson 2002)), water stress (Bosu and Wagner 2014; Gonzáles et al. 2008), and also through mechanical damage (Gonzáles et al. 2008), have differential effects on different herbivores (Karley et al. 2016; Tozin et al. 2017), vary across leaf surfaces (Karabourniotis et al. 1999; Watts and Kariyat 2021b), and have multiple modes of action (Kaur and Kariyat 2020). Studies like this that shows significant variation for trichomes traits (within just 10 varieties) through morphological assays and diversity estimation can further assist in the breeding programs for developing host resistant varieties of tomato, targeting specific pests and their life stages.

We found significant variation for trichome density in our study, not only in major trichome types, but also in the subtypes of trichomes. The importance of trichome density variation has been documented in many studies for its impact not only in biotic stresses (Handley et al. 2005; Valverde et al. 2001) but also in abiotic stresses, such as protection against UV radiations (Karabourniotis et al. 1999), protection against high insolation (Karabourniotis et al. 1999), drought tolerance (Zhang et al. 2020), lowering transpiration water losses (Pérez-Estrada et al. 2000) and resistance to low temperature (Zhang et al. 2020). All these studies show that the trichome density is significantly correlated with plant resistance to biotic and abiotic stresses. Zhang et al. (2020) studied different mutants and cultivars of tomato with varied trichome densities for its relationship to the stress resistance of the plants with treatments of disease, insects, drought and cold. However, the role of density variation of different subtypes of trichomes of tomato in stress resistance needs further investigation, especially from an abiotic stress perspective, for drought management and climate smart crops—to build upon from our work.

The Shannon diversity index and equitability of the trichomes in different varieties was calculated. The equitability of eight out of ten varieties came out as more than 0.7, suggesting that these varieties are very diverse regarding their trichomes. Interestingly, the two varieties having maximum total trichome numbers have the lowest equitability suggesting that the maximum trichome number does not always means maximum diversity. However, the role of this trichome density vs trichome diversity with regard to plant defense mechanisms warrants more experiments.

Taken together, tomato is a very good model for studying the role of trichomes in plant defense as it contains a highly diverse and varied density and dimensions of different trichomes. Future research should explore the importance of density and dimension variation of important subtype of trichomes in tomato. Since tomato is mainly a glandular trichome enriched species, the diversity of volatiles from different cultivars and varieties should be explored in detail. Moreover, the studies regarding trichome-specific transcriptome of tomato which will enable the understanding of metabolic pathways involving in release and sequestration of volatile compounds secreted by the glandular trichomes would be of interest.

## Conclusions

The overall goal of this study was to examine in detail, the morphological characteristics of the trichomes of tomato, *Solanum lycopersicum*, since it is one of the most important domesticated species in Solanaceae. We found enormous variation in not only the number of total trichomes as well as their subtypes, but also in its dimensions, showing intraspecific variations in trichome traits in just 10 commonly cultivated varieties of tomato. This study, we believe will lead to future work in understanding the functional consequences of this intraspecific trichome variations in plant defense against biotic as well as abiotic stresses, by taking into consideration each trichome subtype.

## Supplementary Information

**Additional file 1. Table S1.** Different types of trichomes present in ten varieties of tomato and their line-art representation.

## Data Availability

All raw data will be deposited on public data repository after acceptance of the manuscript.
