# Supplementary material for: Morphological characterization of intraspecific variation for trichome traits in tomato (Solanum lycopersicum)
Source: Bot Stud. 2023 Mar 29;64:7. doi: 10.1186/s40529-023-00370-3 (PMC10060485; doi:10.1186/s40529-023-00370-3)
Supplement: Supplementary file 1 — Additional file 1. Table S1. Different types of trichomes present in ten varieties of tomato and their line-art representation. [file 40529_2023_370_MOESM1_ESM.pptx]

## Slide 1
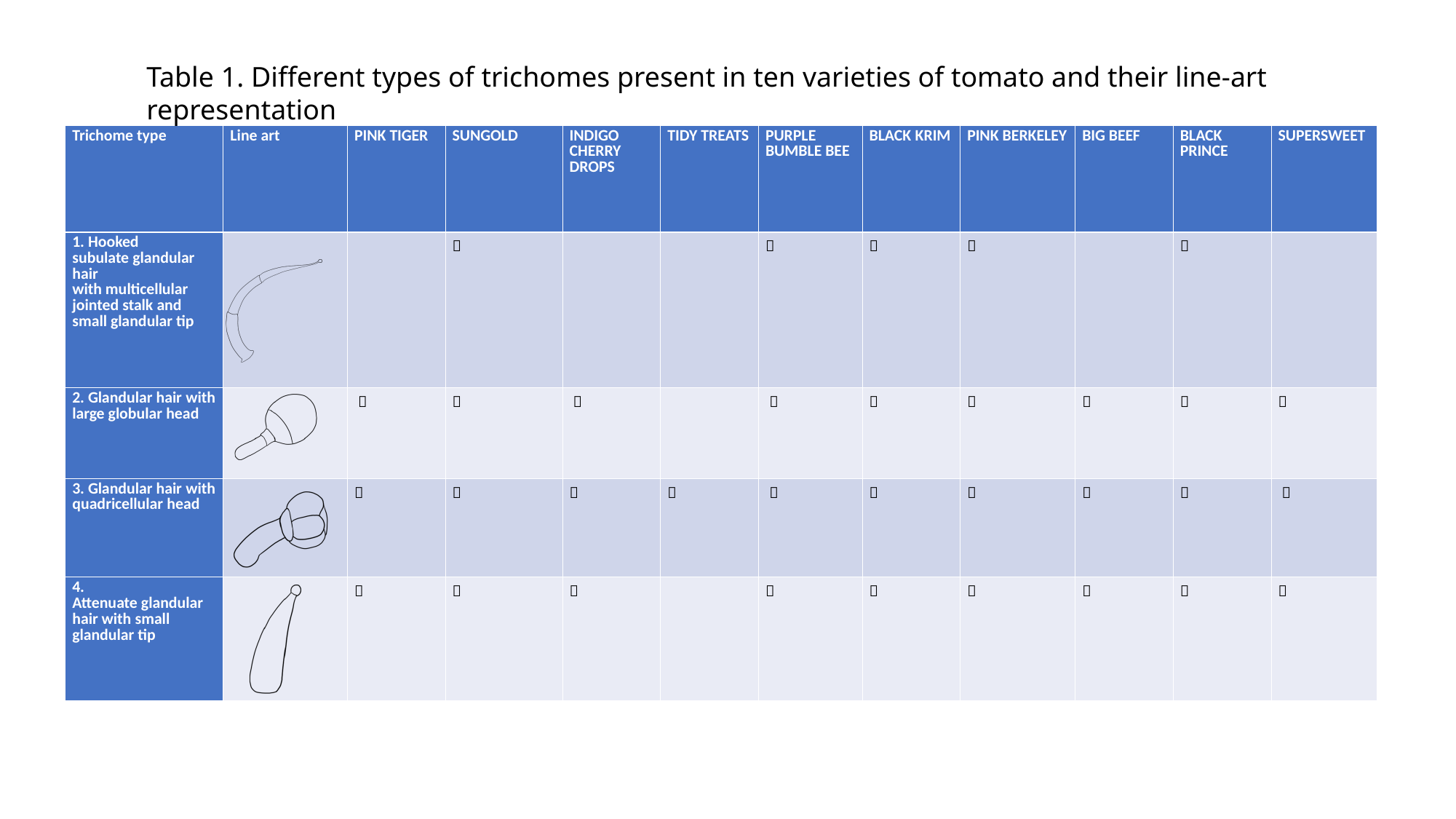

Table 1. Different types of trichomes present in ten varieties of tomato and their line-art representation
| Trichome type​ | Line art  ​ | PINK TIGER​ | SUNGOLD​ | INDIGO CHERRY DROPS | TIDY TREATS​ | PURPLE  BUMBLE BEE​ | BLACK KRIM​ | PINK BERKELEY​ | BIG BEEF​ | BLACK  PRINCE​ | SUPERSWEET​ |
| --- | --- | --- | --- | --- | --- | --- | --- | --- | --- | --- | --- |
| 1. Hooked subulate glandular hair with multicellular jointed stalk and small glandular tip​ | ​ | ​ | ​ | ​ | ​ |  |  |  | ​ |  | ​ |
| 2. Glandular hair with large globular head​ | ​ | ​ |  | ​ | ​ | ​ | ​ | ​ |  |  |  |
| 3. Glandular hair with quadricellular head​ | ​ |  |  | ​ | ​ | ​ | ​ |  |  |  | ​ |
| 4. Attenuate glandular hair with small glandular tip​ | ​ |  |  |  | ​ |  |  |  |  |  |  |

## Slide 2
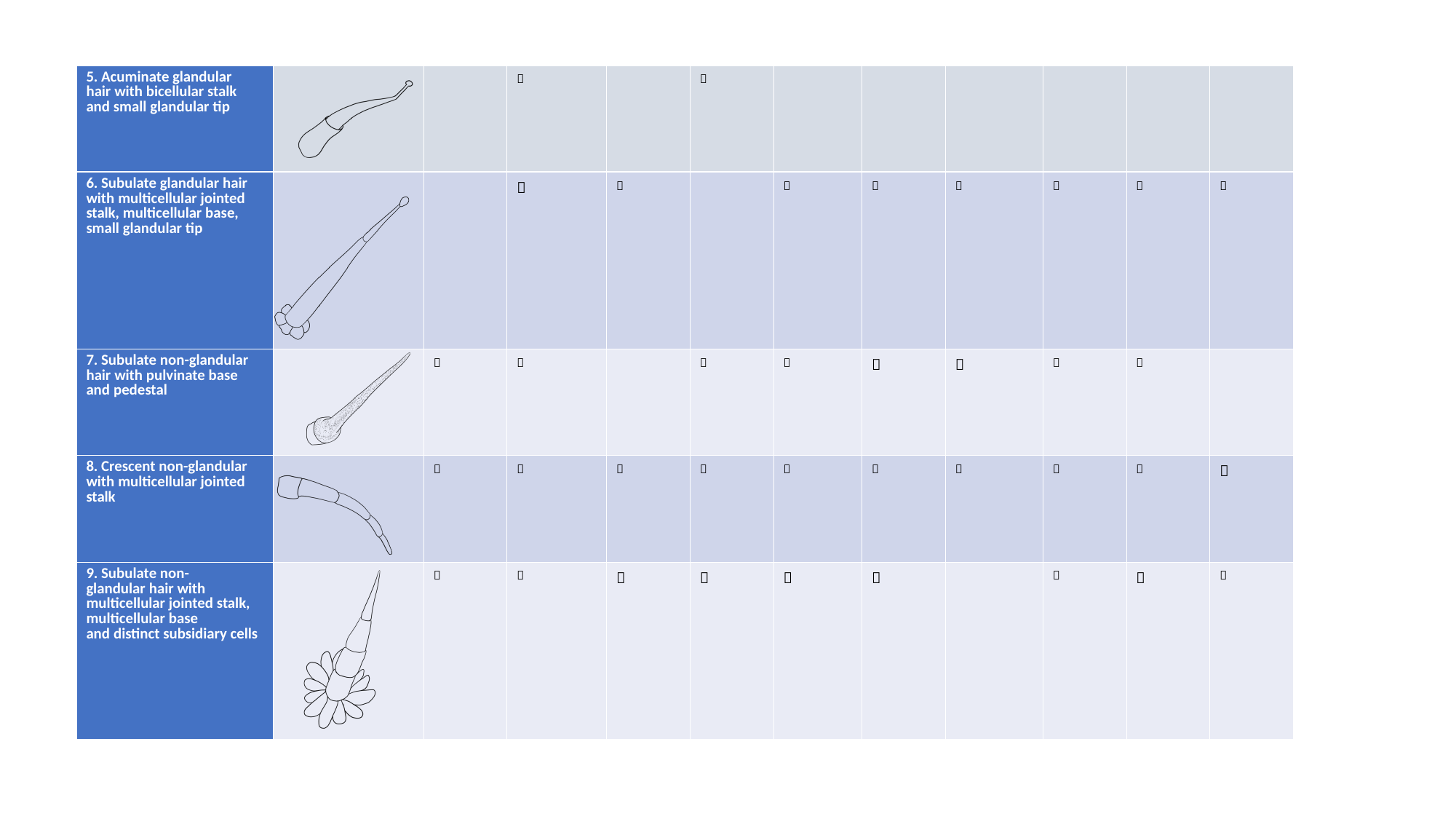

| 5. Acuminate glandular hair with bicellular stalk and small glandular tip​ | ​ | ​ |  | ​ |  | ​ | ​ | ​ | ​ | ​ | ​ |
| --- | --- | --- | --- | --- | --- | --- | --- | --- | --- | --- | --- |
| 6. Subulate glandular hair with multicellular jointed stalk, multicellular base, small glandular tip​ | ​ | ​ | ​ |  | ​ |  |  |  |  |  |  |
| 7. Subulate non-glandular hair with pulvinate base and pedestal​ | ​ |  |  | ​ |  |  | ​ | ​ |  |  | ​ |
| 8. Crescent non-glandular with multicellular jointed stalk​ | ​ |  |  |  |  |  |  |  |  |  | ​ |
| 9. Subulate non-glandular hair with multicellular jointed stalk, multicellular base and distinct subsidiary cells​ | ​ |  |  |  ​ | ​ | ​ |  ​ | ​ |  | ​ |  |
| 6. Acuminate glandular hair with bicellular stalk and small glandular tip​ | ​ | ​ | ​ | ​ | ​ | ​ | ​ | ​ | ​ | ​ | ​ |
| --- | --- | --- | --- | --- | --- | --- | --- | --- | --- | --- | --- |
| 7. Subulate glandular hair with multicellular jointed stalk, multicellular base, small glandular tip​ | ​ | ​ | ​ | ​ | ​ | ​ | ​ | ​ | ​ | ​ | ​ |
| 8. Subulate non-glandular hair with pulvinate base and pedestal​ | ​ | ​ | ​ | ​ | ​ | ​ | ​ | ​ | ​ | ​ | ​ |
| 9. Crescent non-glandular with multicellular jointed stalk​ | ​ | ​ | ​ | ​ | ​ | ​ | ​ | ​ | ​ | ​ | ​ |
| 10. Subulate non-glandular hair with multicellular jointed stalk, multicellular base and distinct subsidiary cells​ | ​ | ​ | ​ | ​ | ​ | ​ | ​ | ​ | ​ | ​ | ​ |
| 6. Acuminate glandular hair with bicellular stalk and small glandular tip​ | ​ | ​ | ​ | ​ | ​ | ​ | ​ | ​ | ​ | ​ | ​ |
| --- | --- | --- | --- | --- | --- | --- | --- | --- | --- | --- | --- |
| 7. Subulate glandular hair with multicellular jointed stalk, multicellular base, small glandular tip​ | ​ | ​ | ​ | ​ | ​ | ​ | ​ | ​ | ​ | ​ | ​ |
| 8. Subulate non-glandular hair with pulvinate base and pedestal​ | ​ | ​ | ​ | ​ | ​ | ​ | ​ | ​ | ​ | ​ | ​ |
| 9. Crescent non-glandular with multicellular jointed stalk​ | ​ | ​ | ​ | ​ | ​ | ​ | ​ | ​ | ​ | ​ | ​ |
| 10. Subulate non-glandular hair with multicellular jointed stalk, multicellular base and distinct subsidiary cells​ | ​ | ​ | ​ | ​ | ​ | ​ | ​ | ​ | ​ | ​ | ​ |
